# Complete Genome Sequences of 20 Nontyphoidal *Salmonella* Isolates from Rwanda

**DOI:** 10.1128/MRA.00016-19

**Published:** 2019-03-21

**Authors:** Maurice Byukusenge, Lingling Li, Mediatrice Uwanyirigira, Venkata R. Vepachedu, Subhashinie Kariyawasam, Manassé Nzayirambaho, Suresh V. Kuchipudi, Bhushan M. Jayarao

**Affiliations:** a Animal Diagnostic Laboratory, Department of Veterinary and Biomedical Sciences, The Pennsylvania State University, University Park, Pennsylvania, USA; b School of Animal Sciences and Veterinary Medicine, University of Rwanda, Nyagatare, Rwanda; c Department of Biology, School of Sciences, University of Rwanda, Kigali, Rwanda; d Division of Clinical Microbiology, Bureau of Laboratories, Pennsylvania Department of Health, Exton, Pennsylvania, USA; e School of Public Health, University of Rwanda, Kigali, Rwanda; University of Maryland School of Medicine

## Abstract

Nontyphoidal Salmonella enterica strains are major foodborne pathogens with global public health importance. Foodborne salmonellosis can be life-threatening, especially in sub-Saharan Africa. We report the complete genome sequences of 20 nontyphoidal Salmonella enterica strains isolated in Rwanda. The reported 20 bacterial chromosomes and 8 plasmids each belong to 1 of 9 nontyphoidal *Salmonella* serotypes.

## Abstract (precis)

Nontyphoidal Salmonella enterica strains are major foodborne pathogens with global public health importance. Foodborne salmonellosis can be life-threatening, especially in sub-Saharan Africa.

## ANNOUNCEMENT

Nontyphoidal *Salmonella* (NTS) serotypes remain a major global public health problem. While foodborne *Salmonella* infections occur worldwide, the most severe and life-threatening infections most frequently occur in low-income countries, such as those in sub-Saharan Africa (1, 2). Only a very few full genome sequences of NTS strains from sub-Saharan Africa are publicly available, which limits the opportunities for in-depth molecular epidemiological studies and outbreak investigations. Here, we report, for the first time, the complete genome sequences of 20 bacterial chromosomes and 8 plasmids belonging to 1 of 9 NTS serotypes isolated from humans, animals, and meat samples from Rwanda. Fecal samples from food production animals (cattle, goats, sheep, and pigs), meat samples (beef, goat, pork, and mutton) from meat retail shops in the Rulindo District of Rwanda, and fecal samples from human patients at Rutongo Hospital and the Remera-Mbogo Health Center (Rulindo, Rwanda) were examined for *Salmonella* spp. The protocols for data collection were approved by the institutional review board (STUDY00007685) and Institutional Animal Care and Use Committee (number 47815) of the Pennsylvania State University and the institutional review board (number 386/CMHS IRB/2017) of the University of Rwanda. *Salmonella* isolation was conducted according to the ISO-6579 standard protocol for isolation and identification of *Salmonella* spp. (3). DNA extraction was performed with the Qiagen Genomic-tip 100/G following the manufacturer’s instructions.

Three different platforms were used for sequencing, the Illumina MiniSeq platform (6 isolates), the Ion Torrent personal genome machine (PGM) system (14 isolates), and the Oxford Nanopore MinION platform (20 isolates). The Nextera DNA Flex library prep kit (Illumina), NEBNext Fast DNA fragmentation and library prep set (New England Biolabs), and Nanopore ligation sequencing kit (SQK-LSK109), with the EXP-NBD103 barcoding kit (Oxford Nanopore Technologies), were used for library preparation for the Illumina, Ion Torrent, and MinION platforms, respectively. The Illumina MiniSeq platform produced an average of about 4 million 150-bp paired-end reads per sample (8 million for both pairs). The Ion Torrent PGM produced an average of about 40,000 single-end reads with an average read length of 310 bp. The MinION platform produced an average of 27,356 reads per sample, with an average length of 20 kb. For all software used, default parameters were used unless otherwise indicated. FastQC version 0.11.8 (4) was used for quality assessment for the Illumina and Ion Torrent reads. Read trimming for quality control was not required. The quality control for MinION reads was performed as described by Wick et al. (5). A hybrid genome assembly approach combining long and short reads was used for *de novo* genome assembly with the Unicycler (version 0.4.7) pipeline (6), with the mode set to “normal” and the options for miniasm+racon bridging and Pilon polishing turned on. The assembled genomes were annotated with the NCBI Prokaryotic Genome Annotation Pipeline (PGAP) (7).

Serotyping of animal and meat *Salmonella* isolates was done at the National Veterinary Services Laboratories (NVSL, Ames, IA) and serotyping of the human isolates at the Pennsylvania State Public Health Laboratory (Exton, PA). The White-Kaufmann-Le Minor scheme and the genome-based serotyping with SeqSero version 1.0 (with raw reads as input) (8) were in agreement for serotype determination for all 20 isolates. The 20 isolates and their serotype and source are summarized in Table 1.

**TABLE 1:** Serotype, source, and SRA and GenBank accession numbers of *Salmonella* isolates from Rwanda

Serotype name	No. of isolates	Isolate source	Type of sample	SRA accession no.	No. of reads		Read length (bp)		Chromosome data			Plasmid data		
					MinION	Illumina/Ion Torrent	MinION	Illumina/Ion Torrent	GenBank accession no.	Size (bp)	GC content (%)	GenBank accession no.	Size (bp)	GC content (%)
Bareilly	1	Pig	Feces	SRS4182820	31,507	503,853	6,899	288	CP034721	4,682,141	52.27			
Moero	4	Human	Feces	SRS4182815^*a*^	19,060	6,184,010	26,233	148	CP034705	4,582,521	52.32			
		Human	Feces	SRS4182814	32,147	452,686	15,554	290	CP034706	4,577,367	52.31			
		Sheep	Feces	SRS4182819	14,762	508,366	33,871	343	CP034722	4,619,542	52.28			
		Cattle	Feces	SRS4182782^*a*^	35,621	6,107,930	14,037	149	CP034718	4,582,522	52.32			
Stanleyville	4	Cattle	Feces	SRS4182818	16,657	368,678	30,019	344	CP034723	4,693,269	52.10	CP034724	63,881	47.08
		Goat	Feces	SRS4182785	33,614	391,653	14,875	296	CP034716	4,643,828	52.12			
		Meat (beef)	Meat	SRS4182787	20,187	739,522	24,769	297	CP034700	4,689,404	52.10	CP034701	63,881	47.08
		Meat (goat)	Meat	SRS4182811	42,848	433,691	11,669	327	CP034703	4,689,056	52.10	CP034704	63,885	47.08
Typhimurium	1	Pig	Feces	SRS4182817^*a*^	13,205	8,008,138	37,866	149	CP034719	4,766,197	52.17	CP034720	93,964	53.13
Karamoja	2	Human	Feces	SRS4182804	19,067	339,763	21,702	285	CP034709	4,764,896	52.20	CP034710	193,610	51.49
		Meat (goat)	Meat	SRS4182788^*a*^	16,104	8,919,500	31,048	149	CP034698	4,768,681	52.21	CP034699	193,615	51.49
Mikawasima	2	Human	Feces	SRS4182801	29,999	434,618	16,667	293	CP034713	4,650,494	52.30	CP034714	126,510	50.66
		Human	Feces	SRS4182790	27,963	275,923	10,073	340	CP034715	4,649,621	52.29			
Waycross	1	Human	Feces	SRS4182807	20,605	328,440	7,410	289	CP034707	4,650,096	52.28	CP034708	82,600	43.08
43:a:1,7	2	Human	Feces	SRS4182796	24,768	313,646	20,188	337	CP034711	4,665,063	52.29			
		Human	Feces	SRS4182798	66,120	332,331	6,701	330	CP034712	4,667,715	52.29			
II 42:r:−	3	Sheep	Feces	SRS4182783^*a*^	11,587	8,424,180	43,155	149	CP034717	4,860,626	51.98			
		Meat (goat)	Meat	SRS4182794^*a*^	31,817	10,627,858	15,715	149	CP034697	4,861,251	51.98			
		Meat (beef)	Meat	SRS4182791	39,482	337,739	12,664	295	CP034702	4,856,831	51.98			

a These samples were sequenced with the Illumina MiniSeq and Oxford Nanopore MinION systems. The other samples were sequenced with the Ion Torrent PGM and MinION systems.

### Data availability.

The complete sequences of the 20 bacterial chromosomes and 8 plasmids have been deposited in GenBank under the accession numbers shown in Table 1. The raw reads have been submitted to the SRA database under the BioProject accession number PRJNA510422.

## References

[1] Ao TT, Feasey NA, Gordon MA, Keddy KH, Angulo FJ, Crump JA 2015 Global burden of invasive nontyphoidal *Salmonella* disease, 2010. Emerg Infect Dis 21:941–949. doi:10.3201/eid2106.140999. PMC4451910 25860298

[2] Majowicz SE, Musto J, Scallan E, Angulo FJ, Kirk M, O’Brien SJ, Jones TF, Fazil A, Hoekstra RM, International Collaboration on Enteric Disease “Burden of Illness” Studies 2010 The global burden of nontyphoidal *Salmonella* gastroenteritis. Clin Infect Dis 50:882–889. doi:10.1086/650733. 20158401

[3] International Organization for Standardization. 2002 General guidance on methods for the Detection of Salmonella, 4th ed. International Organization for Standardization, Geneva, Switzerland.

[4] Andrews S 2010 FastQC: a quality control tool for high throughput sequence data. https://www.bioinformatics.babraham.ac.uk/projects/fastqc/.

[5] Wick RR, Judd LM, Gorrie CL, Holt KE 2017 Completing bacterial genome assemblies with multiplex MinION sequencing. Microb Genom 3:e000132. doi:10.1099/mgen.0.000132. 29177090 PMC5695209

[6] Wick RR, Judd LM, Gorrie CL, Holt KE 2017 Unicycler: resolving bacterial genome assemblies from short and long sequencing reads. PLoS Comput Biol 13:e1005595. doi:10.1371/journal.pcbi.1005595. 28594827 PMC5481147

[7] Tatusova T, DiCuccio M, Badretdin A, Chetvernin V, Nawrocki EP, Zaslavsky L, Lomsadze A, Pruitt KD, Borodovsky M, Ostell J 2016 NCBI Prokaryotic Genome Annotation Pipeline. Nucleic Acids Res 44:6614–6624. doi:10.1093/nar/gkw569. 27342282 PMC5001611

[8] Zhang S, Yin Y, Jones MB, Zhang Z, Deatherage Kaiser BL, Dinsmore BA, Fitzgerald C, Fields PI, Deng X 2015 Salmonella serotype determination utilizing high-throughput genome sequencing data. J Clin Microbiol 53:1685–1692. doi:10.1128/JCM.00323-15. 25762776 PMC4400759

